# Beyond the Rainbow: Suicide, Suicidal Gestures and Self Harm Disparities Among Gender Minorites

**DOI:** 10.1192/j.eurpsy.2025.1170

**Published:** 2025-08-26

**Authors:** J. Parkerson, C. Noureddine, A. Sterchele

**Affiliations:** Psychiatry, Icahn School of Medicine at Mount Sinai, New York, United States

## Abstract

**Introduction:**

Transgender and Gender Non-Conforming (TGNC) individuals are at increased risk of diagnosis with mental disorders, including suicidality and suicidal gestures (Anderson & Ford. Nursing Inquiry 2022; 29). Patients’ psychological distress may be secondary to gender dysphoria; however, the evidence may be unclear.

**Objectives:**

The goal of this review was to compile current evidence to assess a relationship between gender dysphoria and suicidal ideation, suicidal attempt, and self-harming behavior.

**Methods:**

A literature review on PubMed databases was conducted using the search terms “transgender,” “gender non-conforming “suicidality,” “self-harm,” “suicidal gestures,” “child,” “adolescent,” and “youth” in various permutations to assess recent evidence on suicidality and suicidal gestures among TGNC children and adolescents. We also reviewed relationships between gender-dysphoria, social support as a protective factor, and suicidality and/or suicidal gestures among TGNC youths.

**Results:**

A study conducted across 19 states in the United States and large urban school districts found that within the last year, 43.9% of transgender students reported they have seriously considered attempting suicide compared to 20.3% in cis-females and 11.0% cis-males, 39.3% of transgender students reported having a suicide plan compared to <16.0% of cisgender students, and 16.5% of transgender students reported having a suicide attempt requiring medical treatment compared to <2.5% of cisgender students (Garthe et al. Transgend Health 2022; 7 416-422). Another study conducted across three different US cities found higher levels of suicidal ideation and behavior among TGNC youths, compared to their cisgender counterparts (Johns et al. MMWR 2019; 68, 67–71). Also, chosen name use was associated with less suicidal ideation, behavior, and depressive symptoms (Russel et al, J Adolesc Health 2018; 63 503–505). Additionally, a surveillance analysis concluded that TGNC youths reported experiencing higher levels of emotional distress, bullying victimization, risk behaviors (substance use and sexual behavior), and lower levels of protective factors such as internal assets, family connectedness, and feeling safe in their community (Eisenberg et al. J Adolesc Health 2017;61 521–526)

**Conclusions:**

Further research needs to be conducted regarding the relationship between gender dysphoria and suicidality, and the presence of suicidal gestures. However, the current data suggests decreased depressive symptoms, as well as suicidal ideation and behavior associated with increased chosen name usage.

**Disclosure of Interest:**

None Declared

